# Association of Myopia and Genetic Variants of *TGFB2-AS1* and *TGFBR1* in the TGF-β Signaling Pathway: A Longitudinal Study in Chinese School-Aged Children

**DOI:** 10.3389/fcell.2021.628182

**Published:** 2021-04-28

**Authors:** Linjie Liu, Juan He, Xiaoyan Lu, Yimin Yuan, Dandan Jiang, Haishao Xiao, Shudan Lin, Liangde Xu, Yanyan Chen

**Affiliations:** ^1^School of Optometry and Ophthalmology, Wenzhou Medical University, Wenzhou, China; ^2^Eye Hospital, Wenzhou Medical University, Wenzhou, China; ^3^School of Biomedical Engineering, Wenzhou Medical University, Wenzhou, China

**Keywords:** myopia, children, TGF-β signaling pathway, genetic variant, *TGFBR1*, *TGFB2-AS1*

## Abstract

**Background:**

Myopia is a complex multifactorial condition which involves several overlapping signaling pathways mediated by distinct genes. This prospective cohort study evaluated the associations of two genetic variants in the TGF-β signaling pathway with the onset and progression of myopia and ocular biometric parameters in Chinese school-aged children.

**Methods:**

A total of 556 second grade children were examined and followed up for 3.5 years. Non-cycloplegic refraction and ocular biometric parameters were measured annually. Multivariate regression analysis was used to assess the effect of the *TGFBR1* rs10760673 and *TGFB2-AS1* rs7550232 variants on the occurrence and progression of myopia. A 10,000 permutations test was used to correct for multiple testing. Functional annotation of single nucleotide polymorphisms (SNPs) was performed using RegulomeDB, HaploReg, and rVarBase.

**Results:**

A total of 448 children were included in the analysis. After adjustments for gender, age, near work time and outdoor time with 10,000 permutations, the results indicated that the C allele and the AC or CC genotypes of rs7550232 adjacent to *TGFB2-AS1* were associated with a significantly increased risk of the onset of myopia in two genetic models (additive: *P*’ = 0.022; dominant: *P*’ = 0.025). Additionally, the A allele and the AA or AG genotypes of rs10760673 of *TGFBR1* were associated with a significant myopic shift (additive: *P*’ = 0.008; dominant: *P*’ = 0.028; recessive: *P*’ = 0.027). Furthermore, rs10760673 was associated with an increase in axial length (AL) (*P*’ = 0.013, β = 0.03) and a change in the ratio of AL to the corneal radius of curvature (AL/CRC) (*P*’ = 0.031, β = 0.003). Analysis using RegulomeDB, HaploReg, and rVarBase indicated that rs7550232 is likely to affect transcription factor binding, any motif, DNase footprint, and DNase peak.

**Conclusion:**

The present study indicated that rs10760673 and rs7550232 may represent susceptibility loci for the progression and onset of myopia, respectively, in school-aged children. Associations of the variants of the *TGFBR1* and *TGFB2-AS1* genes with myopia may be mediated by the TGF-β signaling pathway; this hypothesis requires validation in functional studies. This trial was registered as ChiCTR1900020584 at www.Chictr.org.cn.

## Introduction

Myopia is one of the most prevalent visual disorders and is very common in school-age children and adolescents, particularly in Asia (Grzybowski et al., 2020). Myopia is generally characterized by axial elongation of the eyeball accompanied with structural changes in the choroid, retina, and sclera. In addition to the economic costs associated with correcting myopia, uncorrected myopia might decrease the quality of life of individuals with associated pathological complications, including glaucoma, retinal detachment, and chorioretinal atrophy, and may lead to permanent loss of vision (Zheng et al., 2013; Ikuno, 2017; Pan et al., 2018; Qian et al., 2018).

The specific etiology of myopia remains unclear. It is generally considered that myopia is caused by a combination of genetic and environmental factors (Morgan et al., 2018; Tedja et al., 2019). The progression of spherical equivalent (SE) and elongation of axial length (AL) are faster in children of myopic parents (Liao et al., 2019). Thus, genetic components play important roles in the pathogenesis of myopia. Recent genome-wide association studies (GWAS) identified an increasing number of common single nucleotide polymorphisms (SNPs) associated with refractive error and myopia. Lin et al. (2009) demonstrated that rs7550232 is one of the susceptibility loci for high incidence of myopia in the Chinese Han population in Taiwan. A recent meta-analysis of GWAS involving 542,934 European participants identified 336 novel genetic loci associated with refractive error. The rs10760673 SNP is one of these genetic loci associated with myopia (Hysi et al., 2020). A study of the ratio of the axial length to corneal radius of curvature (AL/CRC) in subjects of various ages showed opposite effects of the *BMP2* gene in children versus adults, indicating that certain genetic components of refractive error may differ between children and adults (Tideman et al., 2016). Thus, it is important to investigate the relationships between genetic loci identified in adults and myopia in school-aged children.

The rs10760673 variant is located in the intronic region of *TGFBR1* on chromosome 9 (9q22). Transforming growth factor (TGF)-β receptor 1 (TGFBR1), is encoded by the *TGFBR1* gene and participates in the TGF-β signaling pathway, which regulates various physiological and pathological processes, including the control of proliferation and differentiation of mesenchymal cells, wound healing, extracellular matrix (ECM) production, etc. All three TGF-β receptors are downregulated after 1 day of recovery in a tree shrew model of lens-induced myopia, suggesting that these receptors are involved in TGF-β signaling pathway in tree shrew sclera during lens compensation and recovery (Gao et al., 2011). Moreover, the TGF-β signaling pathway participates in ECM remodeling in the sclera and regulates the occurrence and development of myopia on the scleral tissue (Jiang et al., 2017). *TGFBR1* polymorphisms are associated with many diseases, such as Marfan syndrome and cancers (Cario et al., 2018; He et al., 2018). Additionally, a GWAS demonstrated an association of alterations in *TGFBR1* with adult myopia (Hysi et al., 2020). However, the relationships between *TGFBR1* variants and myopia were not investigated in school-aged children.

The rs7550232 variant is located between the *TGFB2* and *TGFB2-AS1* genes. Several studies investigated whether the *TGFB2* gene, which encodes TGF-β2, is associated with myopia (Lin et al., 2009; Jia et al., 2014, 2017). Jia et al. (2014) demonstrated that the concentration of TGF-β2 is positively correlated with AL, suggesting that TGF-β2 is likely to function as a critical factor in axial elongation and myopic shift. However, only a few studies investigated the role of *TGFB2-AS1* in myopia. The *TGFB2-AS1* gene, which encodes a long non-coding RNA (lnc-TGFB2-AS1), is located on chromosome 1 (1q41)^1, 2^, and its transcription is induced by TGF-β through the TGF-β signaling pathway (Papoutsoglou et al., 2019). Recent studies suggested that lncRNAs are differentially expressed in healthy ocular tissues versus eye pathologies, such as neovascularization, proliferative vitreoretinopathy, glaucoma, cataracts, ocular malignancy, or strabismus (Li et al., 2016). Moreover, lncRNA polymorphisms are related to cardiometabolic diseases (Dechamethakun and Muramatsu, 2017). However, the associations of myopia, which is one of the most frequent refractive errors, with mutations in the *TGFB2-AS1* gene were not reported previously.

The present prospective cohort study evaluated the associations of two genetic variants within the TGF-β signaling pathway with the onset and progression of myopia and ocular biometric parameters in Chinese school-aged children.

## Materials and Methods

### Study Population

We used random cluster sampling method to recruit a study population of second grade children at three primary schools in Wenzhou, Zhejiang, China. The participants were enrolled from September 2014 to May 2018. This 3.5-year school-based prospective longitudinal study was associated with the Wenzhou Epidemiology of Refractive Error project. The study protocol was approved by the Eye Hospital of Wenzhou Medical University. Written informed consents were obtained from the parents or guardians of the participants. All study procedures were performed in accordance with the guidelines of the Declaration of Helsinki. All participants underwent ophthalmic evaluations using automatic objective refractometry (non-cycloplegic, RM-800; Topcon Corp., Tokyo, Japan) and measurements of ocular biological structure parameters, including AL and CRC (IOL Master; Carl Zeiss Meditec, Oberkochen, Germany). Time spent on near work and outdoors was obtained from a questionnaire. The SE values were calculated using the following equation: SE = sphere + 0.5 × cylinder. Myopia was defined as a SE of at least −1.00 diopter (D) (Wu et al., 2015b; Ghorbani et al., 2018; Chiang et al., 2020). The definition of myopia is a SE of more than −1.00 D, because refractometry was performed without cycloplegia; hence, the results of refractive measurements could have been artificially decreased in some children due to involuntary accommodation. The CRC was calculated as the average of the steepest and flattest meridians. The AL/CRC ratio was defined as the ratio of the AL to the CRC. Incident myopia was defined as the proportion of children who were non-myopic (initial emmetropes and hyperopes) at baseline but subsequently developed myopia during the follow-up period. The remaining non-myopic children were not diagnosed with myopia at baseline and did not present with myopia at the final follow-up. The annual changes in the refractive error of each eye were determined by calculating the difference in mean SE values at baseline and at follow-up (follow-up value minus the original baseline value) divided by the mean follow-up time in years. A significant myopic shift was defined as a change in SE ≤ −0.50 D/year (Wu et al., 2015a; Hsu et al., 2017). A non-significant myopic shift was defined as a change in SE > −0.50 D/year. The refractive data from both eyes were strongly correlated with each other at all follow-up assessments (Spearman’s ρ = 0.86–0.91); thus, only the data from the right eyes were analyzed.

### SNP Selection and Genotyping

Candidate SNPs were selected based on the database search results and published reports. First, the data on SNPs in the Chinese population were downloaded from the 1000 Genomes Project. Second, tag SNPs were selected using Haploview software from common genetic variations [minor allele frequency (MAF) ≥ 5%] with strong coverage [linkage disequilibrium (LD) *R*^2^ ≥ 0.8]. Finally, two SNPs in two candidate regions were selected for the present study. Details of the selected SNPs are presented in Table 1. We collected saliva to extract DNA and perform target SNP genotyping to avoid invasive blood testing. Genomic DNA was extracted with a DNA extraction kit (Tiangen Biotech Inc., Beijing, China) according to the manufacturer’s instructions.

**TABLE 1:** Information about the rs10760673 (*TGFBR1*) and rs7550232 (*TGFB2-AS1*) SNPs.

SNP	Gene	Chromosome (hg19)	Position	Alleles	MAF	Variant position	HWE *P*-value
rs10760673	*TGFBR1*	9	99116340	G > A	0.394	Intronic variant	0.555
rs7550232	*TGFB2-AS1*	1	218345173	A > C	0.084	Intronic variant	0.497

*MAF, minor allele frequency; information about MAF in East Asian cohorts was obtained from the 1000 Genomes Database. HWE, Hardy-Weinberg equilibrium.*

Single nucleotide polymorphism genotyping was performed using a 48-Plex SNPscan^TM^ kit (cat#: G0104; Genesky Biotechnologies Inc., Shanghai, China) based on double ligation and multiplex fluorescence PCR, as described by Wu in detail (Wu et al., 2019). PCR products were analyzed using an ABI3730XL sequencer. The case or control status of the subjects was masked throughout the analysis. Genotyping of a random duplicated sample was used as an internal control to ensure the quality of the genotyping data, and no genotyping errors were detected for all SNPs. The genotyping success rates were greater than 99%, and the concordance rates were 100% based on 3% duplicate samples.

### Functional Annotation

Functional annotation of the two SNPs was obtained from three functional prediction websites: HaploReg^3^, RegulomeDB^4^, and rVarBase^5^.

RegulomeDB was initially used to identify and compare potential regulatory variants. RegulomeDB (Dong and Boyle, 2019) presents a classification scheme based on the strength of experimental evidence or computational predictions that a variant located in a functional region likely results in a functional consequence. The RegulomeDB provides a score that corresponds to the data available for each individual SNP; lower scores are associated with a wider range of the data supporting functional importance. HaploReg v4.1 was used to annotate the variants and facilitate identification of their potential causal links with disease pathogenesis. HaploReg (Ward and Kellis, 2016) provides functional predictions of potential causal variants and candidate risk loci by systematic mining of comparative, regulatory, and epigenomic annotations. The rVarBase database (version 2.0 of rSNPBase) (Guo et al., 2016) was used to describe the regulatory features of a variant in three dimensions: chromatin states of the surrounding regions, overlapping regulatory elements, and potential target genes.

### Statistical Analysis

Clinical data were analyzed using Statistical Product and Service Solutions software (SPSS version 25, IBM, United States), and genetic data were evaluated using gPLINK version 1.07. Initially, we ensured that all SNPs in the control and case groups passed the Hardy-Weinberg equilibrium (HWE) test. Then, we performed a chi-squared test for three different genetic models (additive, dominant, and recessive models) to determine the distributions of different alleles and genotypes associated with the occurrence and progression of myopia, SE, and ocular parameters and with the corresponding potential genetic models. The additive, dominant, and recessive models were used in genetic analyses to compare the major allele homozygotes with heterozygotes and minor allele homozygotes, major allele homozygotes with heterozygotes + minor allele homozygotes, and major allele homozygotes + heterozygotes with minor allele homozygotes. Normally distributed data are reported as the mean ± standard deviation (SD), and the data that were not normally distributed are presented as the median (P50) and the lower and upper quartiles (P25, P75). Multivariate logistic regression was used to control confounding factors and was adjusted for age, gender, time spent on near work, and time spent outdoors; the results are reported as the estimated odds ratios (ORs) and 95% confidence intervals (CIs). Associations between the SNPs and ocular quantitative traits (including SE, AL, CRC, and AL/CRC) were analyzed using a linear regression model. A *P*-value of <0.05 was considered significant. The Bonferroni correction is too conservative and fails to consider the correlations between the SNPs, which may result in a high false negative rate. Therefore, we used 10,000 permutations test for multiple comparisons in each model, which is considered the gold standard of multiple testing correction in GWAS (Gao et al., 2010; Pahl and Schäfer, 2010). An adjusted *P*-value (*P*’) < 0.05 was considered significant. Finally, generalized multifactor dimensionality reduction (GMDR, GMDR software beta 0.9) was used to identify the genes with gene-gene interactions (GGIs) and to determine the effect of epistasis. The best GGI model was selected based on the trained balance accuracy (TRBA), test balance accuracy (TEBA), and cross-validation consistency (CVC) of the GMDR models.

## Results

### Characteristics of the Study Population

**FIGURE 1:**
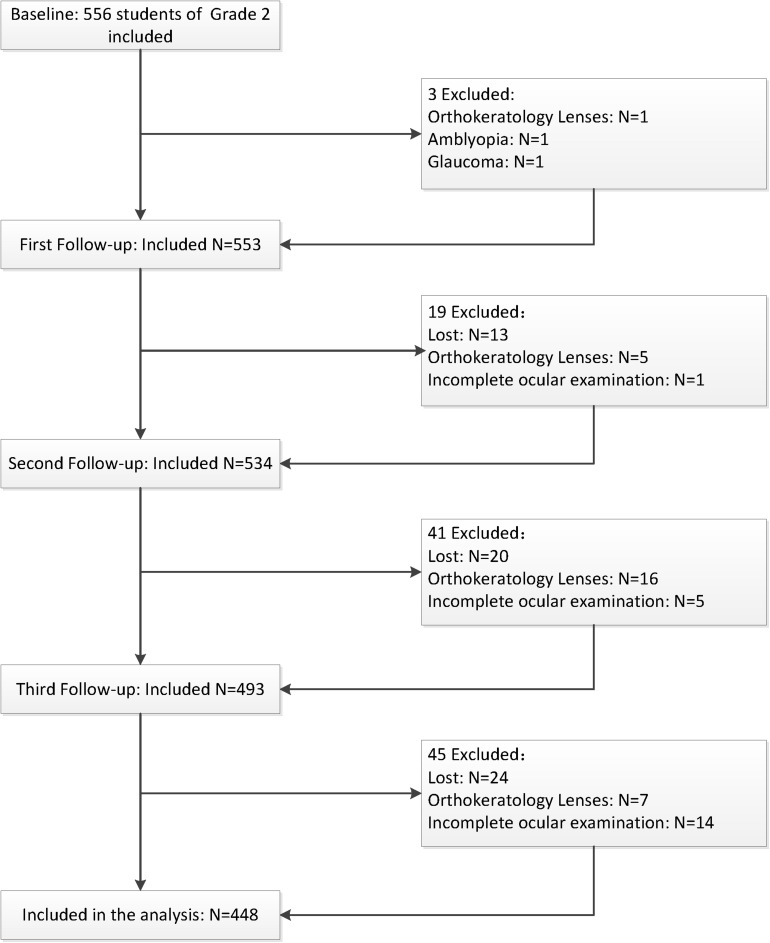
Flow chart of the inclusion and exclusion of the study population.

A total of 556 second grade children were examined at baseline; children who did not have a complete ocular examination (*n* = 20), had ocular diseases or wore orthokeratology lenses (*n* = 31), and did not have the genotyping data due to loss of follow-up (*n* = 57) were excluded during the follow-up stage. Thus, 448 children were included in subsequent analyses. The flow chart of the inclusion and exclusion of the study population is shown in Figure 1. The demographic characteristics and ocular parameters of the participants are described in Table 2. The average age was 7.29 ± 0.46 years. The percentage of males was 54.70% (*n* = 245). As shown in Table 2, the SE of the subjects at baseline was −0.14 D (−0.46, 0.33), and the change in SE was −1.13 D (−2.07, 0.33) during 3.5-year follow-up. Additionally, the baseline AL was 22.96 ± 0.77 mm, with an increase in AL of 1.04 mm (0.70, 1.40). At baseline, the CRC was 7.80 ± 0.26 mm, and the change in CRC was −0.05 mm (−0.09, −0.02). The baseline AL/CRC was 2.95 (2.90, 2.99), and the change in AL/CRC was 0.15 (0.11, 0.21).

**TABLE 2:** Characteristics of the participants.

Variable	
Number	448
Age (years), mean ± SD	7.29 ± 0.46
Gender (boys), N (%)	245 (54.70%)
Baseline SE (D), median (Q1, Q3)	−0.14 (−0.46, 0.33)
Baseline AL (mm), mean ± SD	22.96 ± 0.77
Baseline CRC (mm), mean ± SD	7.80 ± 0.26
Baseline AL/CRC, median (Q1, Q3)	2.95 (2.90, 2.99)
ΔSE (D), median (Q1, Q3)	−1.13 (−2.07, 0.33)
ΔAL (mm), median (Q1, Q3)	1.04 (0.70, 1.40)
ΔCRC (mm), median (Q1, Q3)	−0.05 (−0.09, 0.02)
ΔAL/CRC, median (Q1, Q3)	0.15 (0.11, 0.21)
Near work time (hours/day), median (Q1, Q3)	2.09 (1.00, 3.00)
Outdoor time (hours/day), median (Q1, Q3)	2.12 (1.00, 3.00)

*SE, spherical equivalent refraction; D, diopter; AL, axial length; CRC, corneal radius of curvature. Δ indicates the changes in SE, AL, CRC, and AL/CRC during 3.5-year follow-up.*

### Associations of the SNPs With the Risk of the Onset of Myopia

As shown in Table 1, the distributions of the two SNPs were consistent with HWE (*P* > 0.05). Table 3 lists the allelic and genotypic frequencies of two SNPs in the remaining non-myopic group (*n* = 186) and the incident myopia group (*n* = 212). After adjustment for gender, age, near work activity time, and outdoor time, the rs7550232 C allele variant was associated with increased susceptibility to the onset of myopia in two genetic models (additive: *P* = 0.024, OR = 1.938, 95% CI = 1.092–3.438; dominant: *P* = 0.029, OR = 1.917, 95% CI = 1.069–3.347). In the dominant model, the AC and CC genotypes of rs7550232 were associated with significantly higher risk of the onset of myopia compared with that for the AA genotype. The associations remained significant after 10,000 permutations (additive: *P*’ = 0.022; dominant: *P*’ = 0.025). Significant association of another SNP with incidence of myopia was not detected.

**TABLE 3:** Associations between genetic polymorphisms and the onset of myopia in various genetic models.

Marker	Polymorphisms	Remaining non-myopic		Incident myopia		OR (95% CI)	*P*	*P*’
		No.	%	No.	%			
**rs10760673 (*TGFBR1*)**								
	G	223	60.3	251	59.5			
	A	147	39.7	171	40.5			
Dominant	GG	66	35.7	75	35.5	Reference		
	GA or AA	119	64.3	136	64.5	1.007 (0.664, 1.527)	0.974	0.999
Recessive	GG or GA	157	84.9	176	83.4	Reference		
	AA	28	15.1	35	16.6	1.115 (0.645, 1.928)	0.696	0.704
Additive	–	–	–	–	–	1.034 (0.774, 1.382)	0.819	0.822
**rs7550232 (*TGFB2-AS1*)**								
	A	348	94.5	380	90.5			
	C	20	5.5	40	9.5			
Dominant	AA	164	89.1	171	81.4	Reference		
	AC or CC	20	10.9	39	18.6	1.917 (1.069, 3.347)	0.029*	0.025*
Recessive	AA or AC	184	100	209	99.5	Reference		
	CC	0	0	1	0.5	–	–	–
Additive	–	–	–	–	–	1.938 (1.092, 3.438)	0.024*	0.022*

OR, odds ratio; CI, confidence interval; P, P-value; P’, P-value adjusted by 10,000 permutations. Adjusted for gender, age, near work time, and outdoor time. * Statistically significant at P < 0.05.

### Associations of the SNPs With Myopia Progression

The allele and genotype frequencies of SNPs in the significant myopic shift group (*n* = 145) and the non-significant myopic shift group (*n* = 282) are shown in Table 4. After correction for confounding factors (gender, age, near work time, and outdoor time), binary logistic regression analysis indicated that the *TGFBR1* rs10760673 G > A variant was associated with positive myopia progression. Analysis of the genotype frequencies of the *TGFBR1* rs10760673 G > A polymorphism indicated that a higher fraction of the subjects with the GA or AA genotypes was present in the significant myopic shift group (additive: *P* = 0.008, OR = 1.536, 95% CI = 1.121–2.106; dominant: *P* = 0.029, OR = 1.702, 95% CI = 1.057–2.738; recessive: *P* = 0.027, OR = 1.883, 95% CI = 1.075–3.300). Similar conclusions were achieved after 10,000 permutations (additive: *P*’ = 0.008; dominant: *P*’ = 0.028; recessive: *P* = 0.027). In contrast, no significant differences were observed for another SNP in any models after 10,000 permutations.

**TABLE 4:** Associations between genetic polymorphisms and myopia progression in various genetic models.

Marker	Polymorphisms	Significant myopic shift		Non-significant myopic shift		OR (95% CI)	*P*	*P*’
		No.	%	No.	%			
**rs10760673 (*TGFBR1*)**								
	G	155	53.5	348	62.0			
	A	135	46.5	214	38.0			
Dominant	GG	42	29.0	109	38.8	Reference		
	GA or AA	103	71.0	172	61.2	1.702 (1.057, 2.738)	0.029*	0.028*
Recessive	GG or GA	113	78.0	239	85.1	Reference		
	AA	32	22.0	42	14.9	1.883 (1.075, 3.300)	0.027*	0.027*
Additive	–	–	–	–	–	1.536 (1.121, 2.106)	0.008*	0.008*
**rs7550232 (*TGFB2-AS1*)**								
	A	262	91.0	525	93.7			
	C	26	9.0	35	6.3			
Dominant	AA	118	81.9	246	87.9	Reference		
	AC or CC	26	18.1	34	12.1	1.461 (0.759, 2.816)	0.257	0.256
Recessive	AA or AC	144	100	279	99.6	Reference		
	CC	0	0	1	0.4	–	–	–
Additive	–	–	–	–	–	1.382 (0.732, 2.609)	0.318	0.318

OR, odds ratio; CI, confidence interval; P, P-value; P’, P-value adjusted by 10,000 permutations. A significant myopic shift was defined as ΔSE ≤ −0.50 D/year. Adjusted for gender, age, near work time, and outdoor time. * Statistically significant at P < 0.05.

### Associations of Genetic Variants With Ocular Biometric Parameters

**FIGURE 2:**
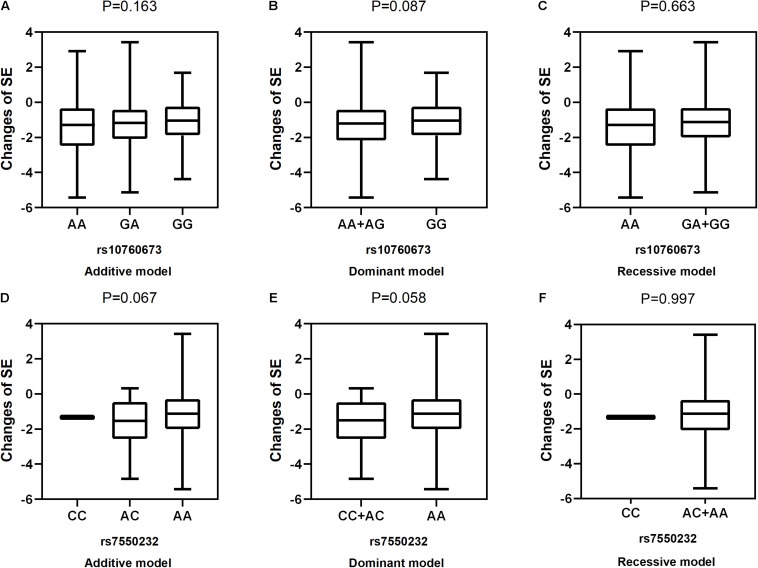
Correlations between the two SNPs and the changes in SE. rs10760673 [**(A)** additive model, *p* = 0.163; **(B)** dominant model, *p* = 0.087; **(C)** recessive model, *p* = 0.663]; rs7550232 [**(D)** additive model, *p* = 0.067; **(E)** dominant model, *p* = 0.058; **(F)** recessive model, *p* = 0.997].

**FIGURE 3:**
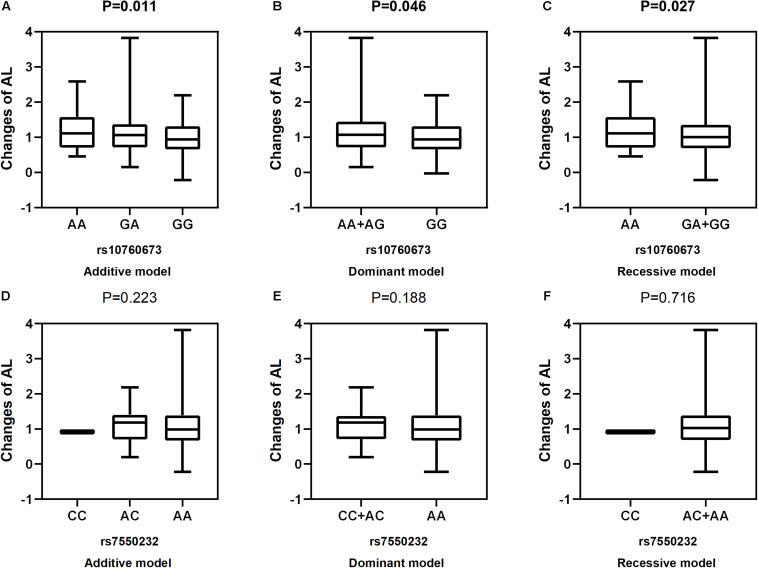
Correlations between the two SNPs and the changes in AL. rs10760673 [**(A)** additive model, *p* = 0.011; **(B)** dominant model, *p* = 0.046; **(C)** recessive model, *p* = 0.027]; rs7550232 [**(D)** additive model, *p* = 0.223; **(E)** dominant model, *p* = 0.188; **(F)** recessive model, *p* = 0.716].

**FIGURE 4:**
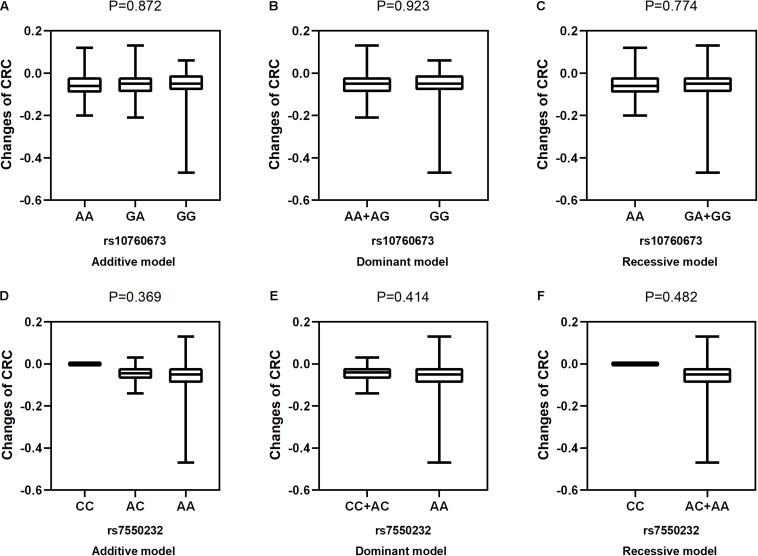
Correlations between the two SNPs and the changes in CRC. rs10760673 [**(A)** additive model, *p* = 0.872; **(B)** dominant model, *p* = 0.923; **(C)** recessive model, *p* = 0.774]; rs7550232 [**(D)** additive model, *p* = 0.369; **(E)** dominant model, *p* = 0.414; **(F)** recessive model, *p* = 0.482].

**FIGURE 5:**
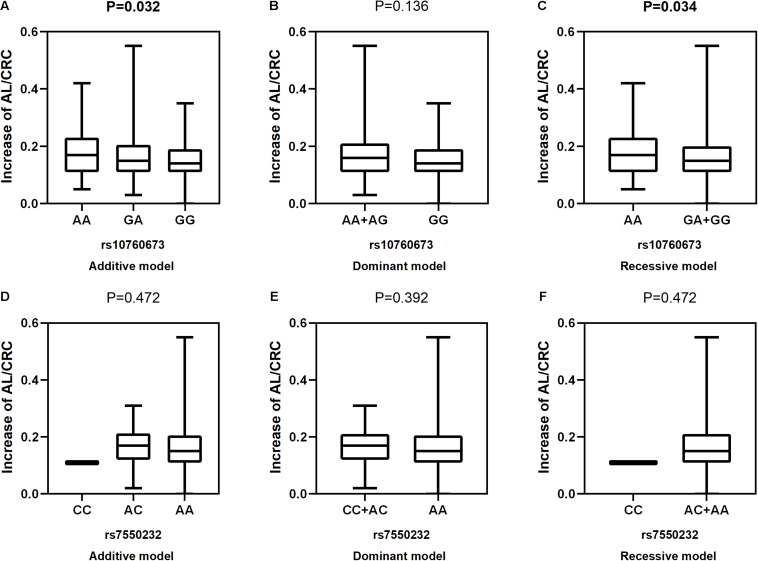
Correlations between the two SNPs and an increase in AL/CRC. rs10760673 [**(A)** additive model, *p* = 0.032; **(B)** dominant model, *p* = 0.136; **(C)** recessive model, *p* = 0.034]; rs7550232 [**(D)** additive model, *p* = 0.472; **(E)** dominant model, *p* = 0.392; **(F)** recessive model, *p* = 0.472].

After adjustment for age, gender, near work activity time, and outdoor time, we performed multivariate linear regression analysis of genetic models. Table 5 and Figures 2–5 show the results of the quantitative trait locus (QTL) analysis. *TGFBR1* rs10760673 was significantly associated with a change in AL (β = 0.03, *P* = 0.011) (Figure 3) and an increase in AL/CRC (β = 0.003, *P* = 0.032) (Figure 5), and was not associated with SE (Figure 2) and CRC (Figure 4). Significant associations of *TGFBR1* rs10760673 with AL were detected in the additive, dominant, and recessive models (*P* = 0.011, *P* = 0.046, *P* = 0.027, respectively). These associations remained significant after 10,000 permutations (*P*’ = 0.013, *P*’ = 0.047, *P*’ = 0.027, respectively). Thus, the A allele and AA genotype were significantly associated with an increase in AL. Children with the AA genotype of rs10760673 had significantly greater AL (1.11 mm) than children carrying the GG (0.94 mm) or GA (1.06 mm) genotypes. Similarly, the AA genotype of *TGFBR1* rs10760673 was associated with an increase in AL/CRC in the additive (*P* = 0.032) and recessive models (*P* = 0.034). Significant differences between the rs10760673 genotypes in these two models remained after 10,000 permutations (*P*’ = 0.031, *P*’ = 0.029, respectively). However, we did not detect any significant associations of *TGFB2-AS1* rs7550232 with the risk for the changes in ocular biometric parameters.

**TABLE 5:** Associations between genetic polymorphisms and the changes in ocular biometric parameters in various genetic models.

Marker	Polymorphisms	N (%)	ΔAL (mm)	*P*	*P*’	ΔAL/CRC	*P*	*P*’
**rs10760673 (*TGFBR1*)**								
Dominant	GG	163 (36.30)	0.94 (0.65, 1.32)			0.14 (0.11, 0.19)		
	GA or AA	180 (63.70)	1.04 (0.71, 1.37)	0.046*	0.047*	0.15 (0.11, 0.21)	0.136	0.139
Recessive	GG or GA	272 (82.90)	0.94 (0.66, 1.31)			0.14 (0.11, 0.19)		
	AA	71 (17.10)	1.11 (0.75, 1.57)	0.027*	0.027*	0.17 (0.11, 0.23)	0.034*	0.029*
Additive	–	–	–	0.011*	0.013*	–	0.032*	0.031*
**rs7550232 (*TGFB2-AS1*)**								
Dominant	AA	380 (85.60)	1.01 (0.68, 1.40)			0.15 (0.11, 0.20)		
	AC or CC	64 (14.40)	1.19 (0.71, 1.37)	0.188	0.184	0.17 (0.12, 0.21)	0.392	0.386
Recessive	AA or AC	443 (99.80)	0.98 (0.67, 1.39)			0.15 (0.11, 0.20)		
	CC	1 (0.20)	0.92 (0.92, 0.92)	0.716	0.752	0.11 (0.11, 0.11)	0.472	0.474
Additive	–	–	–	0.223	0.223	–	0.472	0.473

AL, axial length; CRC, corneal radius of curvature; Δ is the change in AL or AL/CRC during 3.5-year follow-up; P, P-value; P’, P-value adjusted by 10,000 permutations. Adjusted for gender, age, near work time, and outdoor time. *Statistically significant at P < 0.05.

### GGIs Between the Two SNPs

The GMDR model was used to detect the interactions between the two SNPs in the two genes associated with the myopia risk because this model can analyze all possible combinations of the studied polymorphisms. GMDR analysis of the two genetic variants identified the interactions of the genetic polymorphisms associated with incident myopia, which are shown in Table 6. Comparison between the remaining non-myopic group and incident myopia group indicated that rs7550232 adjacent to *TGFB2-AS1* produced the best model with 53.64% TEBA and 10/10 CVC, and no GGIs were identified. The results of GMDR analysis of GGIs associated with significant myopic shift are presented in Table 7. Comparison between the significant myopic shift group and non-significant myopic shift group indicated that the rs10760673 of *TGFBR1* produced the best model with 54.74% TEBA and 10/10 CVC. However, no significant GGIs were identified.

**TABLE 6:** The results of GMDR of multiple loci and genes related to incidence of myopia.

Model	TRBA	TEBA	Sign test (*P*)	CVC
1. *TGFB2-AS1* rs7550232	0.5396	0.5364	6 (0.3770)	10/10
2. Model 1 plus *TGFBR1* rs10760673	0.5446	0.5220	5 (0.6230)	10/10

*GMDR, generalized multifactor dimensionality reduction; TEBA, test balance accuracy; TRBA, trained balanced accuracy; CVC, cross-validation consistency. GMDR was performed to identify genetic variants with gene-gene interactions (GGIs) associated with incident myopia after adjustment for the covariates (age, gender, time spent on near work, and time spent on outdoors). Sign test results show P-value for the significance of the GMDR model.*

**TABLE 7:** The results of GMDR of multiple loci and genes related to a significant myopic shift.

Model	TRBA	TEBA	Sign test (*P*)	CVC
1. *TGFBR1* rs10760673	0.5494	0.5474	7 (0.1719)	10/10
2. Model 1 plus *TGFB2-AS1* rs7550232	0.5586	0.5231	4 (0.8281)	10/10

*GMDR, generalized multifactor dimensionality reduction; TEBA, test balance accuracy; TRBA, trained balanced accuracy; CVC, cross-validation consistency. GMDR was performed to identify genetic variants with gene-gene interactions (GGIs) associated with a significant myopic shift after adjustment for the covariates (age, gender, time spent on near work and time spent on outdoors). Sign test results show P-value for the significance of the GMDR model.*

### Functional Annotation Using Bioinformatics Analysis

According to HaploReg v4.1, rs7550232 was predicted to be located within promoter histone marks in 23 tissues, enhancer histone marks in two tissues (blood and gastrointestinal tract), and 44 DNase hypersensitivity regions and to significantly alter the binding motifs of the EWSR1-FLI1, Irf, and Sp1 transcription factors. Moreover, rs7550232 is located within the binding site of the MAX transcription factor according to the data obtained from the ENCODE project. Additionally, the rs10760673 SNP is located within 17 DNase I hypersensitive regions reported in various cell types; however, histone marks were unavailable. Furthermore, the rs10760673 SNP was predicted to significantly alter the Hsf and TFIIA motifs. The score of rs10760673 provided by RegulomeDB was 4, suggesting that this SNP may be involved in transcription factor binding or DNase peaks. The rs7550232 SNP is likely to influence transcription factor binding any motifs, DNase footprint, and DNase peak; thus, the score equaled 2b, which was classified as having some binding evidence. Additional details are presented in Table 8.

**TABLE 8:** Functional annotation obtained using RegulomeDB, HaploReg, and rVarBase.

SNP	Annotated gene	RegulomeDB	HaploReg v4.1					rVarBase		
		Score	Promoter histone marks	Enhancer histone marks	DNase*	Proteins bound	Motifs changed	Chromatin state**	TF binding	Regulatory SNPs
rs10760673	*TGFBR1*	4	–	–	17	–	Hsf, TFIIA	104	–	60
rs7550232	*TGFB2-AS1*	2b	23 tissue	BLD, GI	44	9	EWSR1-FLI1, Irf, SP1	121	192	75

RegulomeDB score: 2b, TF blinding + any motif + DNase footprint + DNase peak; 4, TF blinding + DNase peak. * : number of tissues with reported DNase evidence of the targeted SNP. ** : number of reported evidence about the chromatin state of the surrounding region, e.g., strong/weak transcription, enhancers, and flanking active transcription start sites. –: No available data. TF, transcription factor; SNP, single nucleotide polymorphism.

## Discussion

Myopia is considered a complex multigenic condition involving several overlapping signaling pathways mediated by the corresponding groups of distinct genes. Therefore, studies of the genetic polymorphisms of myopia-related genes may clarify the mechanism underlying the onset and progression of myopia. Thus, the present study evaluated the associations of two genetic variants with the onset and progression of myopia and with changes in ocular biometric parameters in schoolchildren aged 7–8 years at baseline and during a 3.5-year follow-up and identified several notable patterns of genetic associations. First, the rs10760673 SNP of *TGFBR1* was significantly associated with the progression of myopia (OR = 1.536, *P* = 0.008) and increases in AL and AL/CRC (β = 0.03, *P* = 0.011; β = 0.003, *P* = 0.032, respectively). Second, the rs7550232 SNP adjacent to *TGFB2-AS1* was statistically significantly associated with the occurrence of myopia (OR = 1.938, *P* = 0.024).

### The Association of rs10760673 (*TGFBR1*) With the Progression of Myopia

The results of the present study indicated that rs10760673 was associated with the progression of myopia. A previous GWAS demonstrated that rs10760673 is a susceptibility locus for myopia in adults (Hysi et al., 2020); however, the present study is the first to identify this SNP as a susceptibility locus for myopia progression in school-aged children. Participants with the AA or AG genotypes of rs10760673 tended to have a higher risk of myopia progression. AL is an important indicator of irreversible development of eyeballs in children and adolescents and is related to the formation of myopia (Li et al., 2019). An increase in AL by 1 mm was shown to be associated with 1.74 D and 1.83 D of myopia progression for incipient myopia and persistent myopia, respectively, in primary school-aged children (Ma et al., 2018). Another study showed a 10.72 D shift toward myopia for every 1 unit of an increase in the AL/CRC ratio in Chinese schoolchildren aged 6–12 years (He et al., 2015). Several genes were shown to be associated with AL and AL/CRC; however, previous studies did not report associations between *TGFBR1* and AL or AL/CRC (Cheng et al., 2013; Miyake et al., 2015; Li et al., 2020; Lin et al., 2020; Tang et al., 2020). Therefore, the present 3.5-year cohort study demonstrated that the *TGFBR1* polymorphism was significantly associated with an increase in AL and AL/CRC. The *TGFBR1* polymorphism was not associated with SE in the present study. However, AL and AL/CRC measurements are more precise and less prone to errors than cycloplegic or non-cycloplegic assessments of refraction.

The TGFBR1 protein encoded by the *TGFBR1* gene plays a key role in the TGF-β signaling pathway because the biological effects of TGF-β isoforms are mediated by type I and type II receptors (TGFBR1 and TGFBR2, respectively); the third receptor TGFBR3 functions as an accessory for ligand presentation to TGFBR2. In a tree shrew model of myopia, TGF-β plays an important role in the maintenance of normal morphology and function of the sclera, and the expression of TGF-β isoforms 1, 2, and 3 is downregulated during myopia progression (Jobling et al., 2004). In a tree shrew model of lens-induced myopia, the patterns of differential mRNA expression of TGF-β observed during minus lens compensation (hyperopia) and recovery (myopia) indicated that TGF-β is involved in scleral remodeling (Gao et al., 2011). Additionally, a previous study proposed that the concentrations of TGF-β mRNA and the active form of the TGF-β protein decrease in form-deprived myopic eyes compared with those in the control group. Consequently, TGF-β may mediate the retinal control of AL elongation and influence the progression of myopia (Honda et al., 1996). Thus, the *TGFBR1* gene may influence the progression of myopia and an increase in AL and AL/CRC, which are likely mediated by the TGF-β signaling pathway. According to a previous study, a mutation of the *TGFBR1* gene alters the activity of the TGF-β signaling pathway (Hara et al., 2019). Additionally, functional annotation showed that rs10760673 can change the Hsf motif, which is involved in the regulation of lens and retinal development (Fujimoto et al., 2004; Hawkes et al., 2004). However, specific mechanism of the relationship between the rs10760673 SNP and myopia progression requires additional functional study.

### Association of rs7550232 (*TGFB2-AS1*) With the Occurrence of Myopia

Studies of the pathogenesis of myopia in animal models demonstrated that an increase in eye size facilitated by the remodeling of the sclera is one of the most important etiologies in the progression of myopia (McBrien and Gentle, 2003). The TGF-β signaling pathway was reported to participate in ECM remodeling in the sclera and to regulate the occurrence and development of myopia through the effects of the downstream factors of the pathway on the scleral tissue (Jiang et al., 2017).

The results of the present study demonstrated that the rs7550232 SNP adjacent to *TGFB2-AS1* was significantly associated with the occurrence of myopia. The AA or AC genotypes of the rs7550232 SNP were associated with a higher incidence of myopia in agreement with the results of the previous study in a Han Chinese population in Taiwan (Lin et al., 2009). *TGFB2-AS1* does not encode proteins and regulates transcription, chromatin remodeling, splicing and mRNA translation by scaffolding ribonucleoprotein complexes (Papoutsoglou et al., 2019). Lnc-TGFB2-AS1 counteracts the activity of TGF-β and cooperates with TGF-β signaling to induce or repress the expression of a subset of certain genes (Papoutsoglou et al., 2019). Lnc-TGFB2-AS1 was recently reported to promote ECM deposition via the TGF-β/Smad pathway in human trabecular meshwork cells (Lv et al., 2020). Therefore, we hypothesized that *TGFB2-AS1* may be involved in the onset of myopia by regulating the TGF-β signaling pathway, which has been shown to participate in the ECM remodeling in the sclera during myopia (Gao et al., 2011; Jiang et al., 2017). A study reported that G→T transversion at position +5 of the donor splice site in intron 6 of the androgen receptor gene influences RNA splicing and leads to partial androgen insensitivity syndrome (Sammarco et al., 2000), indicating that intronic mutations located at the donor site of the intron may influence RNA splicing. Function annotation analysis performed in the present study indicated that rs7550232 can bind to transcription factor Sp1, which is a downstream target of TGF-β1, and Sp1 can be detected in human lens epithelial cells (Liu et al., 2016). Furthermore, the expression of Sp1 and collagen I in the scleral tissues decrease with the time of form deprivation myopia at the mRNA and protein levels, suggesting that Sp1 may be involved in the regulation of type I collagen synthesis/degradation during myopic remodeling of the sclera (Jiang et al., 2017). However, additional studies are needed to investigate the biological mechanisms of detected associations between rs7550232 and myopia risk.

The present longitudinal study is the first to investigate the associations of two SNPs with refraction and ocular parameters in Chinese school-aged children, which is the strength of the study. However, the study has some limitations. First, the sample size was relatively small and may be insufficient for the detection of a significant association. Therefore, further larger-scale studies are needed. Second, refractometry was performed without cycloplegia. This approach may lead to overestimation of myopia and under-estimation of hyperopia due to accommodation. However, we defined myopia as −1.00 D, which partially eliminated the errors caused by non-cycloplegic refractometry. Third, both SNPs examined in the study are located in the intronic regions. We annotated the functions of the two SNPs using three functional prediction databases available online; however, further functional studies are needed.

In summary, we demonstrated that the rs10760673 SNP of *TGFBR1* and the rs7550232 SNP adjacent to *TGFB2-AS1* may be new susceptibility loci for the progression and onset of myopia in Chinese school-aged children, respectively. The relationship between the variants of these two genes and myopia may be mediated by the TGF-β signaling pathway, which requires verification in subsequent functional studies.

## Data Availability Statement

The raw data supporting the conclusions of this article will be made available by the authors, without undue reservation.

## Ethics Statement

The studies involving human participants were reviewed and approved by the Ethics Committee of the Eye Hospital of Wenzhou Medical University. Written informed consent to participate in this study was provided by the participants’ legal guardian/next of kin.

## Author Contributions

LL and JH wrote the manuscript. LL, XL, and JH analyzed the data. DJ, YY, and YC assisted with the analyses and participated in constructive discussions. HX and SL helped revise the manuscript. YC and LX contributed to the conception of the study. All authors contributed to the article and approved the submitted version.

## Conflict of Interest

The authors declare that the research was conducted in the absence of any commercial or financial relationships that could be construed as a potential conflict of interest.

## Footnotes

1 https://www.ncbi.nlm.nih.gov/gene/728463

2 https://lncipedia.org/db/transcript/TGFB2-AS1:2

3 http://pubs.broadinstitute.org/mammals/haploreg/haploreg.php

4 http://regulomedb.org/

5 http://rv.psych.ac.cn
